# Leaf N and P stoichiometry in relation to leaf shape and plant size for *Quercus acutissima* provenances across China

**DOI:** 10.1038/srep46133

**Published:** 2017-04-10

**Authors:** Hui Zhang, Xiuqing Yang, Jingyuan Wang, G. Geoff Wang, Mukui Yu, Tonggui Wu

**Affiliations:** 1 East China Coastal Forest Ecosystem Long-term Research Station, Research Institute of Subtropical Forestry, Chinese Academy of Forestry, Fuyang, Zhejiang 311400, PR China; 2 Forestry College, Shanxi Agriculture University, Taigu, Shanxi 030801, PR China; 3 Department of Forestry and Environmental Conservation, Clemson University, Clemson, SC 29634-0317, USA

## Abstract

Plant stoichiometry in relation to the structure and function of biological systems has been investigated at multiple scales. However, few studies have focused on the roles of stoichiometry for a given species. In this study, we determined leaf N and P stoichiometry, leaf shape and plant size in three *Quercus acutissima* common gardens with different climatic and site conditions. In the three common gardens, leaf N and P stoichiometry was significantly correlated with leaf shape and plant size, suggesting that leaf N and P stoichiometry affects the morphological performance of the leaves and stem. The scaling slopes of the relationships between leaf N and P stoichiometry and leaf shape ranged from |0.12| to |1.00|, while the slopes of the relationships between leaf N and P stoichiometry and plant size ranged from |0.95| to |2.66|. These results suggest that non-functional tissues (stem) are more susceptible to leaf nutrition than functional tissues (leaves), and leaf stoichiometry is more important in the construction of non-functional tissues (stem). Between the northernmost and southernmost common gardens, leaf N and leaf width (W), N:P and stem height (H), and N:P and stem diameter (D) showed significant covariations, which indicates that leaf N and W, N:P and plant size exhibit similar plastic responses to environmental change.

Stoichiometry has become a focus of research in ecology and biology in recent years, especially for studies on nutrient cycling and trophic transfer^1^,^2^ in various levels of organization, diverse organisms, and different habitats^3^,^4^. These studies were particularly interested in understanding the nature of trophic interactions^5^ and the pattern of biochemical adjustments as organisms respond to abiotic environmental factors^6^,^7^. By contrast, the roles of stoichiometry were only subjects of a few studies, but these studies suggested that stoichiometry determined the structure and function of biological systems^8^,^9^,^10^,^11^. By examining the stoichiometry at multiple scales^12^,^13^,^14^,^15^, from individual^16^ to community and ecosystem scales^8^,^17^, these studies found that stoichiometry could shift the physiological state of multi-species or the biotic structuring of communities^11^,^18^,^19^,^20^,^21^,^22^. However, the effects of stoichiometry on a single species remain unclear.

Recently, a few studies have noted that stoichiometry could shift the growth form or tissue allocation pattern of an individual plant^23^,^24^,^25^. For example, the increase of the C:N ratio in plant tissue could shift the biomass dominance from photosynthetic to structural tissue^25^. Although these studies have explored the effects of stoichiometry on individual species, the impacts could not be clearly revealed for a given species because both perturbations (e.g., fertilization, warming and elevated atmospheric CO_2_, etc)^11^,^13^,^26^,^27^ and species traits (e.g., growth rate, plant age and plant parts)^28^ can confound the results.

Leaf stoichiometry and individual morphological and physiological traits are indicative of plant strategies, such as plant responses to pressure^29^,^30^,^31^. Thus, studies have increasingly revealed the relationships between these traits^30^,^32^,^33^. For example, leaf nitrogen and phosphorus concentrations are broadly correlated with maximum net photosynthetic rates and leaf mass per unit area (LMA) across thousands of plant species^30^,^32^,^33^. However, most studies have focused on associations between contents of leaf nutrients and physiological traits, such as the photosynthetic rate, specific leaf area (SLA), and leaf longevity^30^, while few studies have focused on relationships between stoichiometry and individual traits, particularly between leaf stoichiometry and morphological traits. Indeed, chemical elements are the basic principles of stoichiometry^5^. Leaf shape and plant size are key aspects of plant morphological performance, which can provide general information about plant growth and investment strategies^34^. Therefore, it is imperative to explore the relationships between leaf stoichiometry and leaf shape and plant size.

Plantations established for provenance testing, where multiple seed sources of a given species were planted in common gardens at the same time, provide a good opportunity for studying the stoichiometry under the same (with one common garden) or different (across several common gardens) environments^35^,^36^,^37^.

We selected three *Quercus acutissima* provenance test plantations established in three common gardens in 2008, with a total of 36 *Q. acutissima* provenances collected across the native range of this species in 13 provinces of China^38^. We then determined the leaf N and P stoichiometry, leaf shape, and plant size for each *Q. acutissima* provenance. We tested two hypotheses: (1) leaf N and P stoichiometry is tightly related to leaf shape and plant size; and (2) leaf stoichiometry co-varies with leaf shape and plant size across two common gardens in different climate conditions.

## Results

### Leaf stoichiometry in relation to leaf shape and plant size in three common gardens

Leaf N showed negative correlations with leaf length (L), width (W) and ground diameter (D) and positive correlations with vein density (VD) and stem height (H) (*P* < 0.05, Table 1). Leaf P was positively correlated with L, leaf-width ratio (L:W), and vein quantity (VQ) and was negatively correlated with vein angle (VA) and H (*P* < 0.05, Table 1). Leaf N:P was negatively associated with L, W, L:W, VQ and D, and positively associated with VD, VA and H (*P* < 0.05, Table 1). Overall, significant relationships were observed between leaf N and P stoichiometry and leaf shape, as well as between leaf N and P stoichiometry and plant size.

The scaling slopes of the relationships between leaf N and P stoichiometry and leaf shape ranged from |0.12| to |1.00|, while the slopes of the relationships between leaf N and P stoichiometry and plant size ranged from |0.95| to |2.66| (Table 1). Most of these slopes were statistically different from |1.00| (*P* < 0.05, Table 1). By contrast, the scaling slope of the relationship between leaf N and W and between leaf N:P and H and D were close to |1.00| (*P* > 0.05, Table 1).

### Covariations in leaf stoichiometry and leaf shape and plant size between the northernmost and the southernmost common gardens

**Figure 1:**
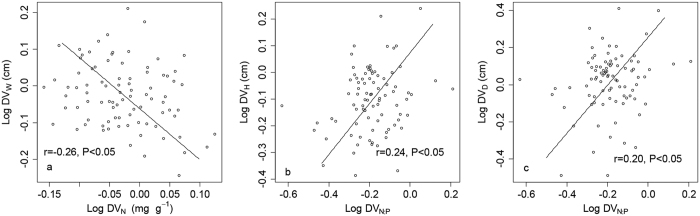
The covariations of leaf stoichiometry, leaf shape, and plant size between the CZ garden and the YF garden. DV_W_ = the difference in leaf width; DV_H_ = the difference in stem height; DV_D_ = the difference in ground diameter; DV_N_ = the difference in nitrogen content; and DV_N:P_ = the difference in the nitrogen-phosphorus ratio.

DV_N_ showed a negative correlation with DV_W_ (*P* < 0.05) (Fig. 1a). The scaling slope of this relationship was statistically lower than −1.00 (*P* < 0.05). DV_N:P_ showed positive correlations with DV_H_ and DV_D_ (Fig. 1b,c). The scaling slopes of the relationships between DV_N:P_ and DV_H_ and DV_D_ were close to 1.00 (*P* > 0.05).

## Discussion

### Leaf stoichiometry in relation to leaf shape and plant size

Our results demonstrate that leaf N and P stoichiometry is significantly correlated with leaf shape and plant size. These relationships suggest that leaf N and P stoichiometry, as the endogenous nutrition, has shaped the morphological performances of leaves and stem, supporting the previous report that N and P frequently limit plant growth^39^. However, we also found that these associations among indices showed huge differences. In this study, leaf P was positively correlated with leaf L and L:W, and these results indicate that leaf P can facilitate the growth of functional tissues (leaf), which is consistent with the prior conclusion that P is the indispensable element to plant growth and development^40^,^41^,^42^, especially to the machinery of cell growth and metabolism^43^. In contrast to the positive relationships, leaf N showed negative correlations with leaf L and W. The results did not correspond with the view that N, as an essential constituent of amino acids, amides, nucleic acids, nucleotides, coenzymes, hexamines, and many other carbon containing compounds^23^, is synthesized to maintain balanced growth^43^,^44^. This may reflect the fact that leaf N is mainly used for the synthesis of more C-compounds, whereas functional N is diluted with the development of leaf growth. In addition, a positive relationship between leaf P and VQ and a negative relationship between leaf P and VA were found because more veins were required to support the longer but narrower leaves based on biomechanical requirements^45^. In addition, the negative relationship between leaf N and VD was mainly due to the smaller leaf size with higher N, which caused the veins to be distributed closely. Furthermore, leaf N:P was significantly correlated with all leaf indices, further suggesting that leaf N and P stoichiometry widely affects leaf growth.

Previous studies have noted that leaf N and P stoichiometry was related to plant size^23^,^46^,^47^. For example, leaf N and P stoichiometry showed quadratic correlations with DBH in a 13-year-old *Machilus pauhoi* stand^24^. In this study, leaf N and N:P showed positive correlations with H and negative correlations with D. These relationships may be due to the individual competitive strategy because the increase of stem axial growth to occupy space above other individuals is beneficial for adequate sunlight^24^. By contrast, leaf P was negatively correlated with H because of more investments of P in the metabolically active fraction of plant mass^11^,^48^. That is, more P is allocated to meet functional or photosynthetic tissue (leaf) growth^11^,^49^ by reducing the non-functional or structural tissues (stem) in response to low P conditions.

The scaling slopes of leaf N and P stoichiometry and leaf shape relationships, excluding the leaf N and W relationship, showed that the changes (increase or decrease) in leaves were far below the changes in leaf N and P stoichiometry. However, this was reversed for leaf N and P stoichiometry and plant size (Table 1). These results show that there is a higher susceptibility to leaf nutrition for non-functional tissues than functional tissues, suggesting that stoichiometry is important to drive the construction of non-functional tissues. In addition, the scaling slopes of relationships between leaf N and W, N:P and H, N:P and D showed isometric changes (Table 1), which suggests that leaf W and stem growth are mainly dependent on leaf N and N:P, respectively.

### The covariations in leaf stoichiometry, leaf shape, and plant size

The relationships between leaf N and P stoichiometry and the growth of individuals are subjected to anthropogenic and environmental perturbations (fertilization^11^, warming^28^) at multiple-scales^8^,^16^,^17^,^25^,^28^. In this study, there were great differences in climate and soil nutrition between the northernmost garden (CZ) and the southernmost garden (YF) (Table 2). Relationships between DV_N_ and DV_W_, DV_N:P_ and DV_H_, and DV_N:P_ and DV_D_ showed significant covariations with leaf N and W and N:P and plant size at these two different gardens. These results indicate that leaf N and W, N:P and plant size have similar plastic responses to environmental changes. Meanwhile, the indices with significant covariations are consistent with those with isometric relationships in the three common gardens, which further supports that there is a high interdependence between leaf stoichiometry and plant size.

The scaling slope of covariation of leaf N and W was significantly lower than −1, which showed that the decreases in DV_N_ were far above the increases in DV_w_. The result supports the viewpoint that leaf W is also highly sensitive to climatic factors^50^. The scaling slopes of covariations of leaf N:P and stem H and D were close to 1, indicating that leaf N:P and plant size vary synchronously with environmental factors.

In summary, leaf N and P stoichiometry was significantly correlated with leaf shape and plant size, suggesting that leaf N and P stoichiometry shaped the outward performance of leaves and stems. Meanwhile, leaf N and P stoichiometry was more important in the construction of non-functional tissues (stem) than functional tissues (leaf). Leaf N and W and N:P and plant size showed significant covariations between the northernmost and the southernmost common gardens, suggesting that leaf N and W and leaf N:P and plant size exhibited similar plastic responses to environmental changes.

## Materials and Methods

### Study area and leaf sample selection

**Figure 2:**
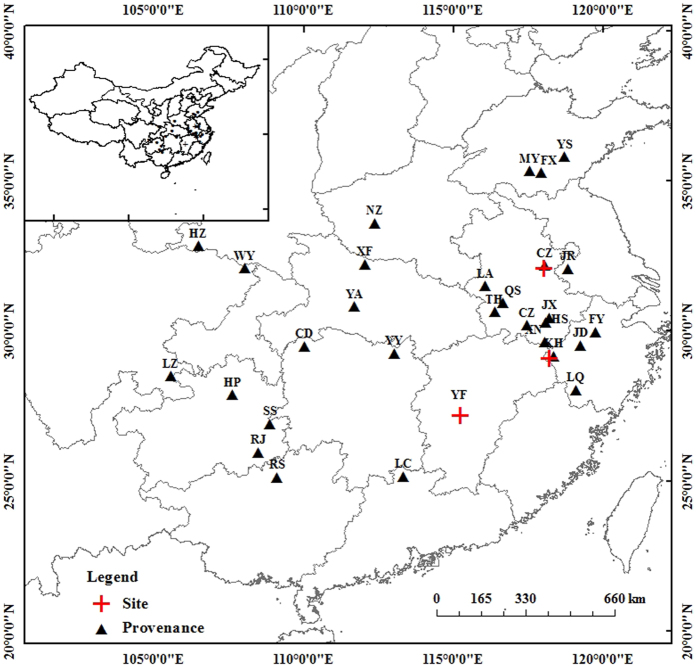
The locations of the 29 investigated *Q. acutissima* provenances (triangles) and the three common gardens (crosses) in Greater China. The map was generated using ArcGIS 10.0 (http://resources.arcgis.com/en/home/).

Thirty-six *Q. acutissima* provenances were planted in three common gardens with different environments: Guanshan Forest Farm in Yongfeng, Jiangxi Province (YF), Kaihua Forest Farm in Kaihua, Zhejiang Province (KH), and Hongyashan Forest Farm in Chuzhou, Anhui Province (CZ) (Fig. 2, Table 2). Table 2 provides the climatic and soil variables for each common garden. Details on provenance selection, seed handling, and seedlings have been previously described^36^. The 36 provenances were planted with a 2 m × 3 m spacing, using a randomized complete block design with six blocks for each provenance and six plants for each block along the mountain slopes in each common garden. Every two blocks as a group were located in the bottom, middle, and top of slopes, respectively^36^. In September 2013, fully expanded and sun-exposed leaves from the 29 provenances that had 100% survival in all three common gardens were selected for the study (Fig. 2). Each sample consisted of 80–100 leaves collected from six trees of each block per provenance per common garden. Samples of the same provenances from the adjacent two blocks from the same location on the mountain slopes were pooled together.

### Measurements

Leaf length (L) and width (W) were measured by rulers, and the length-width ratio (L:W) was calculated by leaf length/width. The leaf vein angle (VA) was determined by protractors for the angle between the midvein and the lateral vein nearest the widest point on the left of leaves, when the leaf apex and back were placed upward; vein quantity (VQ) was counted and vein density (VD) was calculated by leaf vein quantity/(2 × leaf length)^51^,^52^. Samples were oven-dried at 60 °C to a constant weight and then ground finely using a plant sample mill and sieved through a 1-mm mesh screen. The leaf nitrogen concentration (N) was determined for each sample using an auto-analyzer (Kjeltec 2300 Analyzer Unit, Foss, Sweden), and the leaf phosphorus concentration (P) was determined using the standard ammonium molybdate method (reference code GBW08513; General Administration of Quality Supervision, PRC). Plant size (stem height (H) and ground diameter (D)) was measured at the time of leaf sampling.

### Data analysis

Samples from the three common gardens were put together, and we used the data to test whether significant relationships existed between leaf stoichiometry and leaf shape and plant size. To test whether environmental variations affected these relationships, we used the difference (DV) of each index between the northernmost garden and the southernmost garden to analyze the covariations. The DV was determined using the equation:


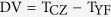


where T_CZ_ is the variable for each index at CZ garden (the northernmost garden), and T_YF_ is the variable for each index at YF garden (the southernmost garden).

The DV calculated for each variable was then log_10_-transformed to normalize the distribution.

To test whether significant relationships and covariations of leaf stoichiometry and leaf shape and plant size existed, we used the standard Pearson correlation test and scatter plots to examine and describe significant relationships and covariations. To test whether an isometric relationship and covariation occurs, we used the equation “log*y* = *a* + *b*(log*x*)” to quantity the relationships and covariations, where *x* is the leaf stoichiometry, *y* is a measure of leaf shape or plant size, *a* is the intercept and *b* is the scaling slope. If the slopes of these relationships are not significantly different from |1.00| (the 95% of confidence interval), the isometric relationships held true. A standardized major axis regression was performed to examine the scaling slope using the ‘smatr’ package. All statistics were analyzed by the R platform (R Development Core Team, 2015) and Excel 2007.

## Additional Information

**How to cite this article:** Zhang, H. *et al*. Leaf N and P stoichiometry in relation to leaf shape and plant size for *Quercus acutissima* provenances across China. *Sci. Rep.* **7**, 46133; doi: 10.1038/srep46133 (2017).

**Publisher's note:** Springer Nature remains neutral with regard to jurisdictional claims in published maps and institutional affiliations.

## Figures and Tables

**Table 1:** The allometric analysis for significant relationships between leaf stoichiometry and leaf shape and plant size.

Y-variable	X-variable	n	r	SMA Slope	lowCI	UppCI	test 1 (*P* value)
N	L	261	−0.31	−0.84	−0.95	−0.75	0.00
N	W	261	−0.27	−1.00	−1.12	−0.89	**0**.**94**
N	VD	261	0.23	0.82	0.73	0.92	0.00
N	H	261	0.17	2.18	1.93	2.46	0.00
N	D	261	−0.21	−2.66	−2.99	−2.36	0.00
P	L	261	0.31	0.48	0.43	0.54	0.00
P	L:W	261	0.40	0.53	0.48	0.60	0.00
P	VA	261	−0.34	−0.15	−0.17	−0.13	0.00
P	VQ	261	0.24	0.46	0.41	0.52	0.00
P	H	261	−0.31	−1.24	−1.39	−1.10	0.00
N:P	L	261	−0.37	−0.37	−0.41	−0.33	0.00
N:P	W	261	−0.13	−0.44	−0.49	−0.39	0.00
N:P	L:W	261	−0.33	−0.41	−0.46	−0.37	0.00
N:P	VD	261	0.17	0.36	0.32	0.41	0.00
N:P	VA	261	0.27	0.12	0.10	0.13	0.00
N:P	VQ	261	−0.22	−0.35	−0.40	−0.31	0.00
N:P	H	261	0.31	0.95	0.85	1.07	**0**.**40**
N:P	D	261	−0.16	−1.16	−1.31	−1.03	**0**.**90**

L = leaf length; W = leaf width; L:W = leaf length:width ratio; VD = leaf vein density; VA = leaf vein angle; VQ = leaf vein quantity; H = stem height; D = ground diameter; N = leaf nitrogen concentration; P = leaf phosphorus concentration; N:P = leaf nitrogen:phosphorus ratio; n = sample size; r = the correlation coefficient; SMA slope = the scaling slope. All data were log_10_-transformed before analysis.

**Table 2:** Climatic and soil features of three common gardens.

Site	LAT	LON	MAT	MAP	Soil		
	°	°	°C	mm	OC	EN	EP
					g kg^−1^	mg kg^−1^	mg kg^−1^
YF	27°19′ N	115°25′ E	18.00	1627.30	18.16	147.29	14.22
KH	29°09′ N	118°23′ E	16.40	1814.00	8.95	109.00	26.76
CZ	32°10′ N	118°04′ E	15.40	1035.50	10.50	166.92	40.37

YF = Guanshan Forest Farm in Yongfeng, Jiangxi Province; KH = Kaihua Forest Farm in Kaihua, Zhejiang Province; CZ = Hongyashan Forest Farm in Chuzhou, Anhui Province. LAT = latitude; LON = longitude; MAT = mean annual temperature; MAP = mean annual precipitation; OC = organic carbon; EN = extractable nitrogen; EP = extractable phosphorus.

## References

[b1] Vanni M. J. Nutrient cycling by animals in freshwater ecosystems. Annu. Rev. Ecol. Evol. Syst. 33, 341–370 (2002).

[b2] Hessen D. O., Ågren G. I., Anderson T. R., Elser J. J. & de Ruiter P. C. Carbon sequestration in ecosystems: the role of stoichiometry. Ecology 85, 1179–1192 (2004).

[b3] Elser J. J. . Biological stoichiometry from genes to ecosystems. Ecol. Lett. 3, 540–550 (2000).

[b4] Ågren G. I. & Weih M. Plant stoichiometry at different scales: element concentration patterns reflect environment more than genotype. New Phytol. 194, 944–952 (2012). 22471439 10.1111/j.1469-8137.2012.04114.x

[b5] Sterner R. W. & Elser J. J. Ecological stoichiometry—the biology of elements from molecules to the biosphere. Princeton University Press, Princeton, New Jersey, USA (2002).

[b6] Reich P. B. & Oleksyn J. Global patterns of plant leaf N and P in relation to temperature and latitude. P. Natl. Acad. Sci. USA 101, 11001–11006 (2004). 10.1073/pnas.0403588101 PMC503733 15213326

[b7] Yuan Z. Y., Chen H. Y. H. & Reich P. B. Global-scale latitudinal patterns of plant fine-root nitrogen and phosphorus. Nat. Commun. 2, 2555–2559 (2010). 10.1038/ncomms1346 21673665

[b8] Yu Q. . Linking stoichiometric homoeostasis with ecosystem structure, functioning and stability. Ecol. Lett. 13, 1390–1399 (2010). 20849443 10.1111/j.1461-0248.2010.01532.x

[b9] Marklein A. R. & Houlton B. Z. Nitrogen inputs accelerate phosphorus cycling rates across a wide variety of terrestrial ecosystems. New Phytol. 193, 696–704 (2012). 22122515 10.1111/j.1469-8137.2011.03967.x

[b10] Sistla S. A. & Schimel J. P. Stoichiometric flexibility as a regulator of carbon and nutrient cycling in terrestrial ecosystems under changes. New Phytol. 196, 68–78 (2012). 22924404 10.1111/j.1469-8137.2012.04234.x

[b11] Sardans J. & Peñuelas J. Trees increase their P:N ratio with size. Global Ecol. Biogeogr. 24, 147–156 (2015). 10.1111/geb.12231 PMC4430815 25983656

[b12] Elser J. J., Dobberfuhl D. R., Mackay N. A. & Schampel J. H. Organism size, life history, and N : P stoichiometry: toward a unified view of cellular and ecosystem processes. BioScience 46, 674–684 (1996).

[b13] Hu S., Chapin F. S. III, Firestone M. K., Field C. B. & Chiariello N. R. Nitrogen limitation of microbial decomposition in a grassland under elevated CO_2_. Nature, 409, 188–191 (2001). 11196641 10.1038/35051576

[b14] Cleveland C. C., Reed S. C. & Townsend A. R. Nutrient regulation of organic matter decomposition in a tropical rain forest. Ecology 87, 492–503 (2006). 16637373 10.1890/05-0525

[b15] Hall E. K. . Linking microbial and ecosystem ecology using ecological stoichiometry: a synthesis of conceptual and empirical approaches. Ecosystems 14, 261–273 (2011).

[b16] Allen A. P. & Gillooly J. F. Towards an integration of ecological stoichiometry and the metabolic theory of ecology to better understand nutrient cycling. Ecol. Lett. 12, 369–384 (2009). 19379132 10.1111/j.1461-0248.2009.01302.x

[b17] Harpole W. S. . Nutrient co-limitation of primary producer communities. Ecol. Lett. 14, 852–862 (2011). 21749598 10.1111/j.1461-0248.2011.01651.x

[b18] Leishman M. R., Thomson V. P. & Cooke J. Native and exotic invasive plants have fundamentally similar carbon capture strategies. J. Ecol. 98, 28–42 (2010).

[b19] Zhu S. D. & Cao K. F. Contrasting cost-benefit strategy between lianas and trees in a tropical seasonal rain forest in southwestern China. Oecologia 16, 591–599 (2010). 10.1007/s00442-010-1579-3 20191291

[b20] Smith V. H. Low nitrogen to phosphorus ratios favor dominance by blue–green algae in lake phytoplankton. Science 221, 669–671 (1983). 17787737 10.1126/science.221.4611.669

[b21] Chapin F. S. III, Vitousek P. M. & Cleve K. V. The nature of nutrient limitation in plant communities. Am. Nat. 127, 48–58 (1986).

[b22] Mack M. C., Schuur E. A. G., Bret-Harte M. S., Shaver G. R. & Chapin F. S. III. Ecosystem carbon storage in arctic tundra reduced by long-term nutrient fertilization. Nature 431, 440–443 (2004). 15386009 10.1038/nature02887

[b23] Niklas K. J. & Cobb E. D. N, P, and C stoichiometry of *Eranthis Hyemalis* (Ranunculaceae) and the allometry of plant growth. Am. J. Bot. 92, 1256–1263 (2005). 21646146 10.3732/ajb.92.8.1256

[b24] Zhang L. L. . Characteristics of leaf carbon, nitrogen and phosphorus stoichiometry in relation to plant size of *Machilus pauhoi*. Chinese J. Appl. Ecol. 26, 1928–1934 (Chinese with English abstract) (2015). 26720927

[b25] Ågren G. I. Stoichimetry and nutrition of plant growth in natural communities. Annu. Rev. Ecol. Evol. S. 39, 153–170 (2008).

[b26] Melillo J. M. . Soil warming, carbon–nitrogen interactions, and forest carbon budgets. P. Nati. Acad. Sci. USA 108, 9508–9512 (2011). 10.1073/pnas.1018189108 PMC3111267 21606374

[b27] Walker M. D. . Plant community responses to experimental warming across the tundra biome. Proc. Nati. Acad. Sci. USA 103, 1342–1346 (2006). 10.1073/pnas.0503198103 PMC1360515 16428292

[b28] Güsewell S. N:P ratios in terrestrial plants: variation and functional significance. New Phytol. 164, 243–266 (2004). 10.1111/j.1469-8137.2004.01192.x 33873556

[b29] Violle C. . Let the concept of trait be functional Oikos 116, 882–892 (2007).

[b30] Wright I. J. . The worldwide leaf economics spectrum. Nature 428, 821–827 (2004). 15103368 10.1038/nature02403

[b31] Roelofsen H. D., van Bodegom P. M., Kooistra L. & Witte J. M. Predicting leaf traits of herbaceous species from their spectral characteristics. Ecol. Evol. 4, 706–719 (2014). 24683454 10.1002/ece3.932 PMC3967897

[b32] Westoby M. & Wright I. J. Land-plant ecology on the basis of functional traits. Trends Ecol. Evol. 21, 261–268 (2006). 16697912 10.1016/j.tree.2006.02.004

[b33] Farnsworth E. J. & Ellison A. M. Prey availability directly affects physiology, growth, nutrient allocation and scaling relationships among leaf traits in 10 carnivorous plant species. J. Ecol. 96, 213–221 (2008).

[b34] Wang L. L. . C:N:P stoichiometry and leaf traits of halophytes in an arid saline environment, northwest China. Plos One 10, e0119935 (2015). 25798853 10.1371/journal.pone.0119935 PMC4370893

[b35] Matyas C. Modeling climate change effects with provenance test dada. Tree Physiol. 14, 797–804 (1994). 14967649 10.1093/treephys/14.7-8-9.797

[b36] Zhang H., Guo W., Wang G., Yu M. & Wu T. Effect of environment and genetics on leaf N and P stoichiometry for *Quercus acutissima* across China. Eur. J. Forest Res., doi: 10.1007/s10342-016-0973-8 (2016).

[b37] Thomson A. M. & Parker W. H. Boreal forest provenance tests used to predict optimal growth and response to climate change. 1. Jack pine. Can. J. Forest Res. 38, 157–170 (2008).

[b38] Liu Z., Fang S., Liu D., Yu M. & Tang L. Influence of thinning time and density on sprout development, biomass production and energy stocks of sawtooth oak stumps. Forest Ecol. Manag. 262, 299–306 (Chinese with English abstract) (2011).

[b39] Xu X. L. . Nutrient limitation of alpine plants: Implications from leaf N : P stoichiometry and leaf δ^15^N. J. Plant Nutr. Soil Sc. 177, 378–387 (2014).

[b40] Bieleski R. L. Phosphate pools, phosphate transport, and phosphate availability. Annu. Rev. Plant Physi. 24, 225–252 (1973).

[b41] Raghothama K. G. Phosphate acquisition. Ann. Rev. Plant Physio. Plant Mol. Biol., 50, 665–693 (1999). 10.1146/annurev.arplant.50.1.665 15012223

[b42] Vance C. P., Uhde-Stone C. & Allan D. L. Phosphorus acquisition and use: critical adaptations by plants for securing a nonrenewable resource. New Phytol. 157, 423–447 (2003). 10.1046/j.1469-8137.2003.00695.x 33873400

[b43] Ågren G. I. The C:N:P stoichiometry of autotrophs: theory and observations. Ecol. Lett. 7, 185–191 (2004).

[b44] Vrede T., Dobberfuhl D. R., Kooijman S. A. L. M. & Elser J. J. Fundamental connections among organism C:N:P stoichiometry, macromolecular composition, and growth. Ecology 85, 1217–1229 (2004).

[b45] Roth-Nebelsick A., Uhl D., Mosbrugger V. & Kerp H. Evolution and function of leaf venation architecture: A review. Ann. Bot. 87, 553–566 (2001).

[b46] Liu F. D. . Plant size effects on the relationships among specific leaf area, leaf nutrient content, and photosynthetic capacity in tropical woody species. Acta Oecol. 36, 149–159 (2010).

[b47] Li X. L. . Linking nutrient strategies with plant size along a grazing gradient: evidence from *Leymus chinensis* in a natural pasture. J. Integr. Agr., doi: 10.1016/S2095-3119(15)61171-6 (2015).

[b48] Sardans J. & Peñuelas J. Tree growth changes with climate and forest type are associated with relative allocation of nutrients, especially phosphorus, to leaves and wood. Global Ecol. Biogeogr. 22, 494–507 (2013).

[b49] Peri P. L., Gargaglione V. & Pastur G. M. Dynamics of above- and below-ground biomass and nutrient accumulation in an age sequence of *Nothofagus antarctica* forest of Southern Patagonia. Forest Ecol. Manag. 233, 85–99 (2006).

[b50] Wu L. L., Kang H. Z., Zhuang H. L. & Liu C. J. Variations of *Quercus variabilis* leaf traits in relation to climatic factors at regional scale. Chinese J. Ecol. 29, 2309–2316 (Chinese with English abstract) (2010).

[b51] Hashizume H., Suo Z., Lee J. H. & Okada S. The basic study on breeding of Oak (II) – Variations in leaf and fruit traits of *Quercus dentata* Thunb., *Q. serrata* Thunb., *Q. mongolica* Fischer var. *grosseserrata* Rehder et Wilson and their intermediate types. J. Jap. Forestry Soc. 105, 325–328 (1994).

[b52] Li Y., Cui J. & Su Y. Specific leaf area and leaf dry matter content of some plants in different dune habitats. Acta Ecol. Sin. 25, 301–304 (Chinese with English abstract) (2005).

